# Development and validation of a LightGBM-based diagnostic model for acute-on-chronic liver failure in patients with liver cirrhosis: a multicenter retrospective study in an HBV-predominant cohort

**DOI:** 10.3389/fmed.2026.1922075

**Published:** 2026-10-27

**Authors:** Manni Xiao, Xiao Zhang, Juntao Tan, Qingzhu Tan, Siying Li, Rui Li, Qiqin Luo, Xiaoni Zhong

**Affiliations:** 1 Department of Pulmonary and Critical Care Medicine, Affiliated Banan Hospital of Chongqing Medical University, Chongqing, China; 2 Department of Health Statistics, School of Public Health, Chongqing Medical University, Chongqing, China; 3 College of Artificial Intelligence Medicine, Chongqing Medical University, Chongqing, China; 4 Department of Medical Records and Statistics, Affiliated Banan Hospital of Chongqing Medical University, Chongqing, China; 5 School of Mathematics and Statistics, Hubei Minzu University, Enshi, China; 6 Department of Infectious and Liver Diseases, Affiliated Banan Hospital of Chongqing Medical University, Chongqing, China

**Keywords:** acute-on-chronic liver failure, cirrhosis, diagnostic model, machine learning, multicenter

## Abstract

**Background:**

Acute-on-chronic liver failure (ACLF) is a severe and often fatal complication in patients with liver cirrhosis. Because its clinical manifestations in the early stage are often masked by the chronic liver disease, timely identification and risk stratification of ACLF are critical for improving clinical outcomes. Therefore, the present study aimed to develop a model for timely identification and risk stratification of ACLF.

**Methods:**

In this multicenter retrospective study, data were collected from seven tertiary hospitals in Southwest China, including 17,567 patients with cirrhosis between 2012 and 2023. Feature selection was performed using Least Absolute Shrinkage and Selection Operator (LASSO) regression. Multiple machine learning algorithms, including Light Gradient Boosting Machine (LightGBM), were developed and compared for ACLF prediction. Model performance was assessed and validated using the area under the receiver operating characteristic curve (AUC–ROC), area under the precision–recall curve (AUC–PR), calibration curves, and decision curve analysis. The TreeSHAP framework was applied for model interpretation.

**Results:**

A total of 13,490 eligible patients with cirrhosis were included, of whom 1,250 (9.27%) developed ACLF. The cohort was randomly divided into a training set (*n* = 9,758) and a test set (*n* = 4,182). Among the evaluated algorithms, the LightGBM model demonstrated the best predictive performance. In the training set, the LightGBM model yielded an AUROC of 0.960, an AUPRC of 0.727, an accuracy of 0.882, a sensitivity of 0.954, a specificity of 0.875 and an F1 score of 0.593. In the test set, the corresponding values were 0.948, 0.655, 0.866, 0.912, 0.861, and 0.549, respectively. SHAP analysis identified the nine most important predictive features, ranked by their contribution to the model: international normalized ratio, total bilirubin, prealbumin, hepatitis B virus infection, alanine aminotransferase, platelet count, total bile acid, hepatic encephalopathy and alcoholic liver disease.

**Conclusion:**

A LightGBM-based diagnostic model incorporating nine key clinical variables was successfully developed. This model facilitates timely identification and risk stratification of ACLF in patients with cirrhosis, which may support clinical decision-making at presentation and targeted clinical interventions.

## Introduction

Liver cirrhosis (LC) is a chronic progressive liver disease characterized by irreversible degeneration of hepatocytes following long-term and repeated liver injury, resulting in severe impairment of liver function (1). In 2017, an estimated 122 million individuals were living with LC worldwide, and its prevalence continues to rise (2). LC has contributed substantially to the increasing burden of liver-related mortality globally and remains a major public health challenge (3). As the disease progresses, patients with cirrhosis frequently develop various complications, among which acute-on-chronic liver failure (ACLF) is one of the most common and life-threatening (4). The clinical course of ACLF often closely resembles fluctuations in cirrhotic decompensation because of the masking effects of underlying chronic liver disease and the overlap in clinical manifestations. This combination of diagnostic difficulty and insidious onset contributes to the high mortality associated with ACLF. ACLF is a clinical syndrome characterized by the rapid deterioration of liver function in patients with underlying chronic liver disease following an acute precipitating event, resulting in high short-term mortality (5, 6). Epidemiological studies have demonstrated substantial geographical variation in the etiology of ACLF. HBV infection is the predominant cause in the Asia-Pacific region, accounting for approximately 60%–70% of cases, whereas alcoholic liver disease (ALD) is the leading etiology in Western countries, responsible for more than 50% of cases (7). Currently, the management of ACLF remains focused on treating precipitating factors and providing organ-supportive care. Although novel therapeutic approaches, including immunotherapy and cellular therapies, have emerged in recent years, none have yet achieved a transformative improvement in clinical outcomes (8).

Liver transplantation remains the only curative treatment for ACLF; however, its efficacy is substantially reduced in patients with multiple organ failure (9, 10). Studies have shown that prophylactic interventions administered before the onset of ACLF, such as antiviral therapy, anti-infective therapy, and artificial liver support, can effectively delay disease progression. In patients with pre-acute-on-chronic liver failure (pre-ACLF), these measures have been associated with a reduction in 90-day mortality from 58% to 35% (11, 12). Reported ACLF cases have risen over the past decade, but this trend may partly reflect evolving definitions, diagnostic practices, and regional criteria differences. Consequently, epidemiological trends in ACLF remain difficult to interpret. An important study by Wang et al. identified the precipitating factors that trigger ACLF in patients with HBV-LC (13). Moreover, ACLF is characterized by rapid, often abrupt, clinical deterioration, and serial changes in clinical parameters provide time-critical information for bedside decision-making (14). Therefore, accurate disease assessment and risk stratification of ACLF at clinical presentation are crucial for enabling timely and individualized therapeutic interventions.

Machine learning (ML) facilitates disease prediction by processing large volumes of clinical data to uncover complex patterns, thereby enabling accurate prediction of adverse clinical outcomes (15). With the rapid growth of healthcare big data, an increasing number of ML studies have focused on liver diseases (16). However, most existing models have been developed and validated for complications of LC, whereas relatively few have specifically addressed ACLF. Furthermore, many of these studies rely on a single ML algorithm and conventional feature-selection methods, which may limit their predictive performance and generalizability (17, 18). A major challenge in the clinical application of ML models is their limited interpretability, as they are often regarded as “black-box” models that provide little insight into the underlying decision-making process. To address this issue, SHapley Additive exPlanations (SHAP), a game-theory-based interpretability framework, can quantify the contribution of each feature to model predictions (19), thereby enhancing transparency and facilitating interpretation of model outputs.

Herein, we aimed to develop a model for the timely identification and risk stratification of ACLF in patients with LC using multiple machine learning algorithms. Feature variables were selected using logistic regression in combination with the Least Absolute Shrinkage and Selection Operator (LASSO) method. To overcome the limitations of previous studies, including limited model interpretability and reliance on single-center data, we used a multicenter dataset to systematically develop and compare six machine learning models. Model interpretability was further enhanced by applying SHAP to visualize and explain model predictions. The findings of this study are expected to support the early identification of ACLF and facilitate more informed clinical decision-making.

## Materials and methods

### Data source

This multicenter retrospective study enrolled 17,567 patients with cirrhosis who were admitted to seven tertiary hospitals in Chongqing, China, between January 2012 and December 2023. Patients were eligible if they met any of the following criteria: (1) histological findings consistent with a diagnosis of cirrhosis; (2) diagnosis of cirrhosis based on the detection of esophageal or gastric varices by gastroscopy; (3) imaging evidence (ultrasound, computed tomography [CT], or liver stiffness measurement [LSM]) suggestive of cirrhosis or portal hypertension; or (4) the presence of at least two of the following laboratory abnormalities: platelet count <100 × 10⁹/L, serum albumin <35 g/L, international normalized ratio (INR) > 1.3, or AST-to-platelet ratio index (APRI) > 2 in adults (20). Patients were excluded if they met any of the following criteria: (1) age <18 years or >80 years; (2) pregnancy; (3) presence of malignancy; (4) human immunodeficiency virus (HIV) infection; or (5) an overall clinical data missing rate exceeding 25%. The technical workflow of the study is illustrated in Figure 1.

**Figure 1:**
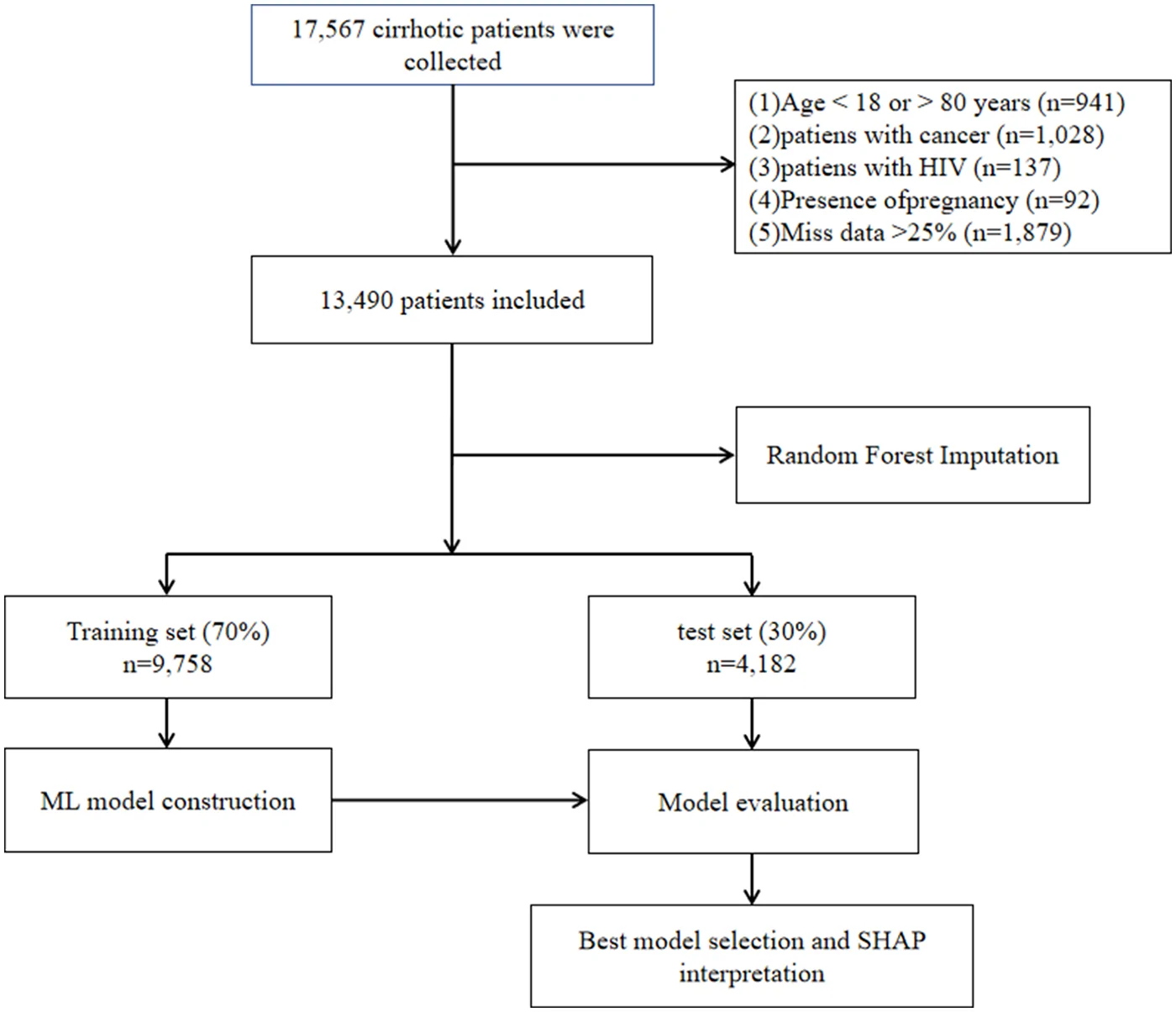
Flowchart of patient selection and model development.

The study protocol was approved by the Ethics Committee of the Affiliated Banan Hospital of Chongqing Medical University (Approval Number: BNLLKY2024047). Given the retrospective observational design of the study, the ethics committee waived the requirement for informed consent. Nevertheless, the study was conducted in strict accordance with ethical principles. In addition, the study methodology adhered to the Transparent Reporting of a Multivariable Prediction Model for Individual Prognosis or Diagnosis (TRIPOD) guidelines.

### Data collection

The variables included in this study were selected based on a comprehensive evaluation of the etiology, pathophysiology, disease progression, and treatment of LC and ACLF. A total of 32 variables were ultimately included, including demographic characteristics, clinical history, and laboratory parameters. Demographic and clinical variables included age, sex, current medical history, past medical history, smoking history, and alcohol consumption history. Laboratory parameters included indicators of liver, renal, and coagulation function, complete blood count, and electrolyte status. Liver function-related variables included alanine aminotransferase (ALT), total bilirubin (TBIL), direct bilirubin (DBIL), alkaline phosphatase (ALP), gamma-glutamyl transferase (GGT), total bile acids (TBA), international normalized ratio (INR), total protein (TP), and prealbumin (PA). Renal function indicators included creatinine (Cr) and uric acid (UA). Additional laboratory variables included potassium (K), sodium (Na), chloride (Cl), magnesium(Mg), hemoglobin (Hb), white blood cell count (WBC), and platelet count (PLT). Comorbidities and cirrhosis- related complications included hepatitis B virus (HBV) infection, hepatitis C virus (HCV) infection, alcoholic liver disease (ALD), autoimmune hepatitis (AIH), portal hypertension (PHT), hepatic encephalopathy (HE), esophagogastric variceal bleeding (EGVB), ascites, hypertension, and diabetes mellitus.

### Outcome measures

The present study aimed to develop a diagnostic model for ACLF in patients with cirrhosis. The study outcome was ACLF at presentation, defined using a modified version of the 2019 APASL criteria: acute hepatic injury characterized by a total bilirubin level >5 mg/dL and an INR ≥1.5 (21). To prevent circular reasoning between predictors and the outcome, ascites and hepatic encephalopathy, standard components of the full APASL definition, were excluded from the outcome definition and evaluated strictly as predictor variables.

All patients who developed ACLF met the diagnostic criteria at admission, and their clinical parameters were collected as part of the diagnostic evaluation. For non-ACLF patients, baseline parameters collected at admission were used. Accordingly, the model estimates the probability of concurrent ACLF at presentation.

### Statistical methods

Here, the dataset was randomly divided into training and testing sets in a 7:3 ratio. Missing values in feature variables (≤25%) were imputed using a random forest-based imputation method. The Kolmogorov–Smirnov (K-S) test was used to assess the normality of the data. Normally distributed variables were expressed as mean ± standard deviation (SD) and compared between groups using the t-test. Non-normally distributed variables were presented as medians (interquartile ranges, IQRs) and compared using the rank-sum test. Categorical variables were expressed as *n* (%) and compared using the chi-square test.

Univariate logistic regression was first performed for initial screening, followed by LASSO regression for feature selection. Ten-fold cross-validation was used to determine the optimal *λ* value based on the one-standard error rule, yielding a parsimonious set of features. To minimize multicollinearity, only variables with a Variance Inflation Factor (VIF) < 5 and tolerance >0.1 were retained for multivariable modeling to identify independent risk factors. Based on these selected features, six machine learning models were constructed, including extreme gradient boosting (XGBoost), light gradient boosting machine (LightGBM), gradient boosting decision tree (GBDT), decision tree (DT), random forest (RF), and logistic regression (LR). To reduce the risk of overfitting, hyperparameters were optimized using Bayesian optimization combined with ten-fold cross-validation. Model performance was primarily evaluated using the area under the receiver operating characteristic curve (AUC-ROC). To address class imbalance, class weight adjustment was applied during model training, which substantially improved sensitivity and overall predictive performance. In addition, model performance was assessed using the area under the precision–recall curve (PR-AUC), which better reflects the trade-off between precision and recall in imbalanced datasets. To select the optimal model, multiple performance metrics were considered, including sensitivity (Sens), specificity (Spe), accuracy (Acc), positive likelihood ratio (PLR), negative likelihood ratio (NLR), diagnostic odds ratio (DOR), and F1 score. The predictive effectiveness and clinical utility of the models were further evaluated using calibration curves and decision curve analysis (DCA), respectively.

Moreover, we used SHAP with the TreeSHAP algorithm to transparently interpret model predictions. Summary plots were generated to illustrate overall feature importance and the direction of feature effects across the entire dataset. Force plots were used to explain individual predictions by decomposing the contribution of each feature for specific cases. Together, these methods provide both global and local interpretability of the models. Statistical significance was defined as a two-sided *P*-value < 0.05. All statistical analyses were performed using Python (version 3.9.1) and R (version 4.5.1).

## Results

### Baseline characteristics

A total of 13,490 patients were included in this study according to the predefined inclusion and exclusion criteria, of whom 1,250 were diagnosed with ACLF. Patients were randomly assigned to a training set (70%, *n* = 9,758; 868 ACLF and 8,890 non-ACLF cases) and a testing set (30%, *n* = 4,182; 382 ACLF and 3,800 non-ACLF cases). The baseline characteristics of the study population are presented in Table 1.

**Table 1:** Baseline characteristics comparison between training set and test set.

Variable	Total (*n* = 13,490)	Training set (*n* = 9,758)	Test set (*n* = 4,182)	p
AIH	1,237 (8.87%)	865 (8.86%)	372 (8.90%)	0.979
ALD	1,838 (13.19%)	1,290 (13.22%)	548 (13.10%)	0.874
Ascites	977 (7.01%)	677 (6.94%)	300 (7.17%)	0.643
DM	1,981 (14.21%)	1,393 (14.28%)	588 (14.06%)	0.759
HBV	7,696 (55.21%)	5,314 (54.46%)	2,382 (56.96%)	0.107
HCV	587 (4.21%)	402 (4.12%)	185 (4.42%)	0.440
HE	642 (4.61%)	446 (4.57%)	196 (4.69%)	0.798
Hypertension	1,524 (10.93%)	1,067 (10.93%)	457 (10.93%)	0.993
PHT	2,001 (14.35%)	1,412 (14.47%)	589 (14.08%)	0.569
Cl	104.40 (101.00,107.40)	104.40 (101.00,107.40)	104.40 (101.00,107.30)	0.894
K	3.89 (3.58,4.20)	3.89 (3.59,4.20)	3.89 (3.58,4.20)	0.480
Mg	0.79 (0.74,0.85)	0.79 (0.74,0.85)	0.79 (0.74,0.86)	0.342
Na	139.30 (136.40,141.70)	139.30 (136.40,141.70)	139.40 (136.50,141.70)	0.953
PLT	79.00 (51.00,128.00)	78.00 (50.00,128.00)	79.73 (52.00,130.00)	0.177
WBC	4.47 (3.11,6.40)	4.47 (3.11,6.43)	4.46 (3.10,6.31)	0.909
Age < 65	3,034 (21.76%)	2,163 (22.17%)	871 (20.83%)	0.083
Drinking	5,680 (40.75%)	3,991 (40.90%)	1,689 (40.39%)	0.586
Male	9,472 (67.95%)	6,624 (67.88%)	2,848 (68.10%)	0.815
Smoking	5,604 (40.20%)	3,933 (40.31%)	1,671 (39.96%)	0.715
ALP	111.00 (81.00,159.00)	110.00 (81.00,159.00)	111.70 (81.00,158.00)	0.650
ALT	33.00 (20.20,65.00)	33.00 (20.30,65.00)	33.00 (20.11,65.00)	0.897
Cr	66.69 (56.30,80.92)	66.40 (56.09,80.50)	67.10 (56.80,81.80)	0.078
DBIL	12.19 (6.60,31.30)	12.20 (6.50,31.16)	12.16 (6.60,32.10)	0.919
EGVB	2,231 (16.00%)	1,570 (16.09%)	661 (15.81%)	0.694
GGT	57.70 (28.00,129.00)	58.00 (28.00,129.00)	57.00 (28.00,129.00)	0.932
Hb	110.00 (88.00,129.00)	110.00 (88.00,129.00)	110.00 (88.00,129.00)	0.477
INR	1.29 (1.13,1.54)	1.29 (1.13,1.55)	1.29 (1.13,1.54)	0.672
PA	87.00 (54.00,134.42)	87.50 (54.00,134.38)	87.00 (54.00,134.69)	0.848
TBA	31.50 (11.90,79.50)	30.92 (11.70,78.47)	32.90 (12.48,81.20)	0.149
TBIL	25.80 (15.59,53.80)	25.84 (15.60,54.20)	25.80 (15.50,53.10)	0.687
TP	66.30 (59.70,72.80)	66.30 (59.70,72.79)	66.10 (59.60,72.80)	0.367
UA	293.00 (219.90,374.73)	292.85 (220.03,375.50)	293.30 (219.70,373.25)	0.806

ALT, alanine aminotransferase (U/L); TBIL, total bilirubin (*μ*mol/L); DBIL, direct bilirubin (μmol/L); ALP, alkaline phosphatase (U/L); GGT, gamma-glutamyl transferase (U/L); TBA, total bile acids (μmol/L); INR, international normalized ratio; TP, total protein (g/L); PA, prealbumin (mg/L); Cr, creatinine (μmol/L); UA, uric acid (μmol/L); K, potassium (mmol/L); Na, sodium (mmol/L); Cl, chloride (mmol/L); Mg, magnesium (mmol/L); Hb, hemoglobin (g/L); WBC, white blood cell count (×10⁹/L); PLT, platelet count (×10⁹/L); HBV, hepatitis B virus; HCV, hepatitis C virus; ALD, alcoholic liver disease; AIH, autoimmune hepatitis; PHT, portal hypertension; HE, hepatic encephalopathy; EGVB, esophagogastric variceal bleeding; DM, diabetes mellitus.

Among patients with liver cirrhosis, those with ACLF were characterized by a higher proportion of individuals aged >65 years and a greater proportion of males. In terms of comorbidities, the proportions of patients with HBV, ALD, HE, and ascites were higher, whereas the proportions of patients with HCV, PHT, AIH, and hypertension were lower. Significantly lower levels were also observed for liver function parameters (DBIL, INR, PA, TP), electrolytes (Mg, Na), renal function marker (UA), and platelet count (PLT).

### Feature variables

Based on the training set, univariate logistic regression identified 28 variables significantly associated with ACLF, including age, sex, drinking, smoking, ALT, DBIL, GGT, INR, PA, TBA, TBIL, TP, PLT, WBC, Cl, K, Mg, Na, AIH, ALD, ascites, DM, HBV, HCV, HE, hypertension, PHT, and UA (Table 2)**.**

**Table 2:** Baseline characteristics comparison between ACLF and Non-ACLF patients

Variable	Total (*n* = 9,758)	Non-ACLF (*n* = 8,890)	ACLF (*n* = 868)	p
AIH	865 (8.86%)	812 (9.1%)	53 (6.1%)	0.003
ALD	1,290 (13.22%)	1,128 (12.7%)	162 (18.7%)	<0.001
Ascites	677 (6.94%)	586 (6.6%)	91 (10.5%)	<0.001
DM	1,393 (14.28%)	1,296 (14.6%)	97 (11.2%)	0.006
HBV	5,314 (54.46%)	4,658 (52.4%)	656 (75.6%)	<0.001
HCV	402 (4.12%)	388 (4.4%)	14 (1.6%)	<0.001
HE	446 (4.57%)	280 (3.1%)	166 (19.1%)	<0.001
Hypertension	1,067 (10.93%)	995 (11.2%)	72 (8.3%)	0.009
PHT	1,412 (14.47%)	1,351 (15.2%)	61 (7.0%)	<0.001
Cl	104.40 (101.00,107.40)	104.5 (101.2,107.5)	102.6 (98.5,106.2)	<0.001
K	3.89 (3.59,4.20)	3.9 (3.6,4.2)	3.8 (3.5,4.2)	0.003
Mg	0.79 (0.74,0.85)	0.8 (0.7,0.8)	0.8 (0.7,0.8)	0.001
Na	139.30 (136.40,141.70)	139.4 (136.7,141.8)	137.4 (133.6,140.4)	<0.001
PLT	78.00 (50.00,128.00)	80.0 (51.0,130.0)	66.5 (40.0,107.2)	<0.001
WBC	4.47 (3.11,6.43)	4.4 (3.1,6.3)	5.4 (3.8,8.3)	<0.001
Age < 65	2,163 (22.17%)	2,067 (23.3%)	96 (11.1%)	<0.001
Drinking	3,991 (40.90%)	3,583 (40.3%)	408 (47.0%)	<0.001
Male	6,624 (67.88%)	5,976 (67.2%)	648 (74.7%)	<0.001
Smoking	3,933 (40.31%)	3,503 (39.4%)	430 (49.5%)	<0.001
ALP	110.00 (81.00,159.00)	108.0 (80.0,157.0)	134.5 (99.0,171.0)	0.061
ALT	33.00 (20.30,65.00)	32.0 (20.0,58.0)	77.0 (30.8,302.8)	<0.001
Cr	66.40 (56.09,80.50)	66.3 (56.0,80.2)	67.1 (56.6,84.6)	0.808
DBIL	12.20 (6.50,31.16)	10.9 (6.2,23.9)	100.2 (36.9,196.4)	<0.001
EGVB	1,570 (16.09%)	1,444 (16.2%)	126 (14.5%)	0.187
GGT	58.00 (28.00,129.00)	57.0 (28.0,130.0)	61.9 (29.0,112.2)	<0.001
Hb	110.00 (88.00,129.00)	111.0 (88.0,129.0)	108.0 (90.0,127.2)	0.579
INR	1.29 (1.13,1.55)	1.3 (1.1,1.5)	2.1 (1.8,2.6)	<0.001
PA	87.50 (54.00,134.38)	94.0 (59.9,139.3)	43.0 (28.0,59.1)	<0.001
TBA	30.92 (11.70,78.47)	27.5 (10.8,67.0)	128.6 (59.2,189.0)	<0.001
TBIL	25.84 (15.60,54.20)	23.6 (14.8,44.0)	149.5 (67.6,271.1)	<0.001
TP	66.30 (59.70,72.79)	66.7 (60.2,73.1)	61.6 (56.4,67.9)	<0.001
UA	292.85 (220.03,375.50)	298.0 (227.4,378.6)	221.4 (146.8,317.9)	<0.001

ALT, alanine aminotransferase (U/L); TBIL, total bilirubin (μmol/L); DBIL, direct bilirubin (μmol/L); ALP, alkaline phosphatase (U/L); GGT, gamma-glutamyl transferase (U/L); TBA, total bile acids (μmol/L); INR, international normalized ratio; TP, total protein (g/L); PA, prealbumin (mg/L); Cr, creatinine (μmol/L); UA, uric acid (μmol/L); K, potassium (mmol/L); Na, sodium (mmol/L); Cl, chloride (mmol/L); Mg, magnesium (mmol/L); Hb, hemoglobin (g/L); WBC, white blood cell count (×10⁹/L); PLT, platelet count (×10⁹/L); HBV, hepatitis B virus; HCV, hepatitis C virus; ALD, alcoholic liver disease; AIH, autoimmune hepatitis; PHT, portal hypertension; HE, hepatic encephalopathy; EGVB, esophagogastric variceal bleeding; DM, diabetes mellitus.

Variables with *P* < 0.05 in the univariate logistic regression analysis were subsequently subjected to LASSO regression for feature selection (Figure 2). After multicollinearity assessment (VIF <5, tolerance >0.1), a total of nine feature variables were retained, including INR, TBIL, PA, HBV, ALT, PLT, TBA, HE, and ALD**.** The Pearson correlation heatmap of these selected features is shown in Figure 3.

**Figure 2:**
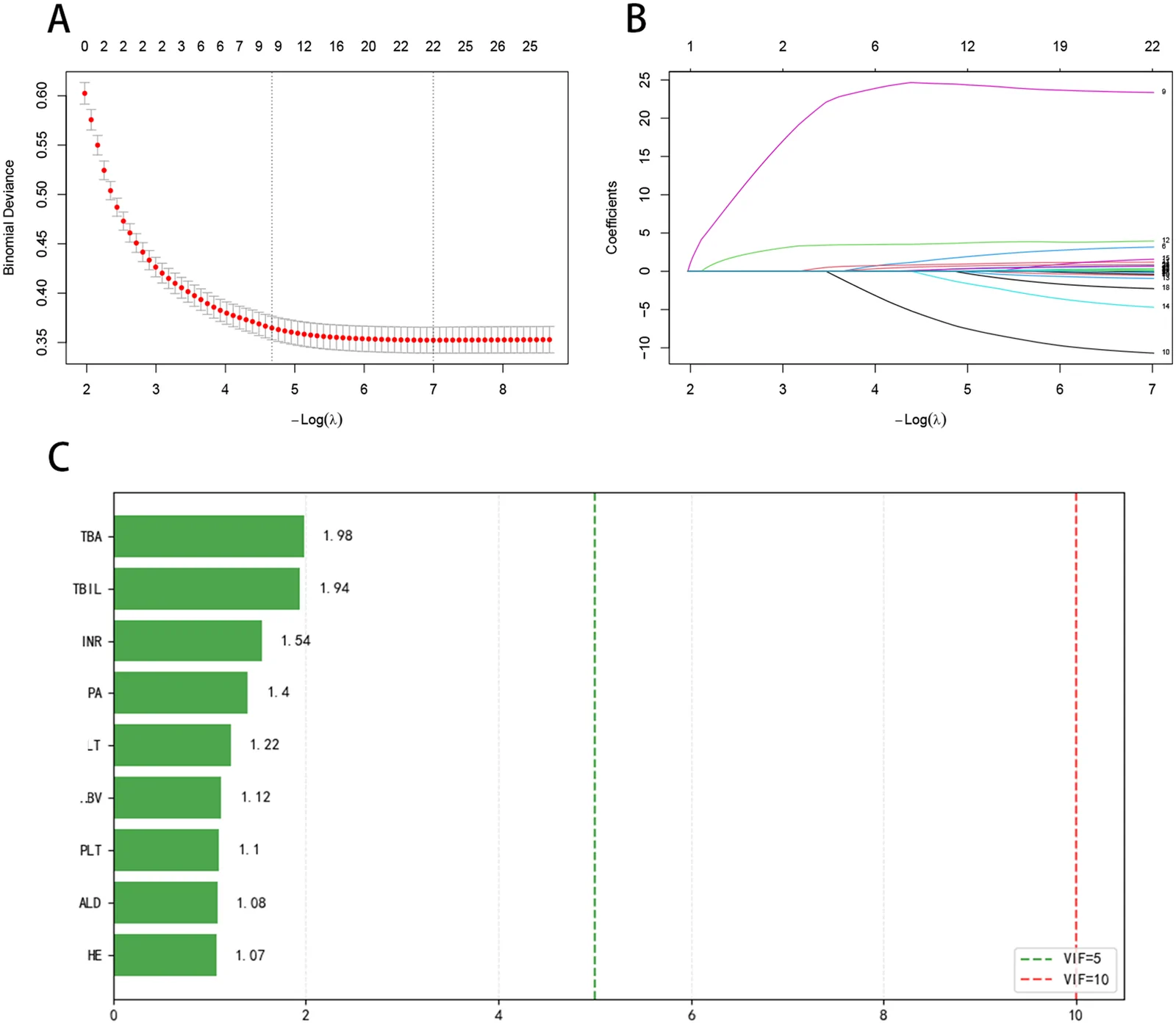
Variable screening based on LASSO regression. **(A)** depicts the coefficient variation paths. **(B)** illustrates the selection of the optimal *λ* value through cross-validation. **(C)** Bar plot of variance inflation factor (VIF) for assessing multicollinearity among independent variables. The green dashed line denotes VIF = 5, suggesting potential multicollinearity; the red dashed line marks VIF = 10, indicating severe multicollinearity.

**Figure 3:**
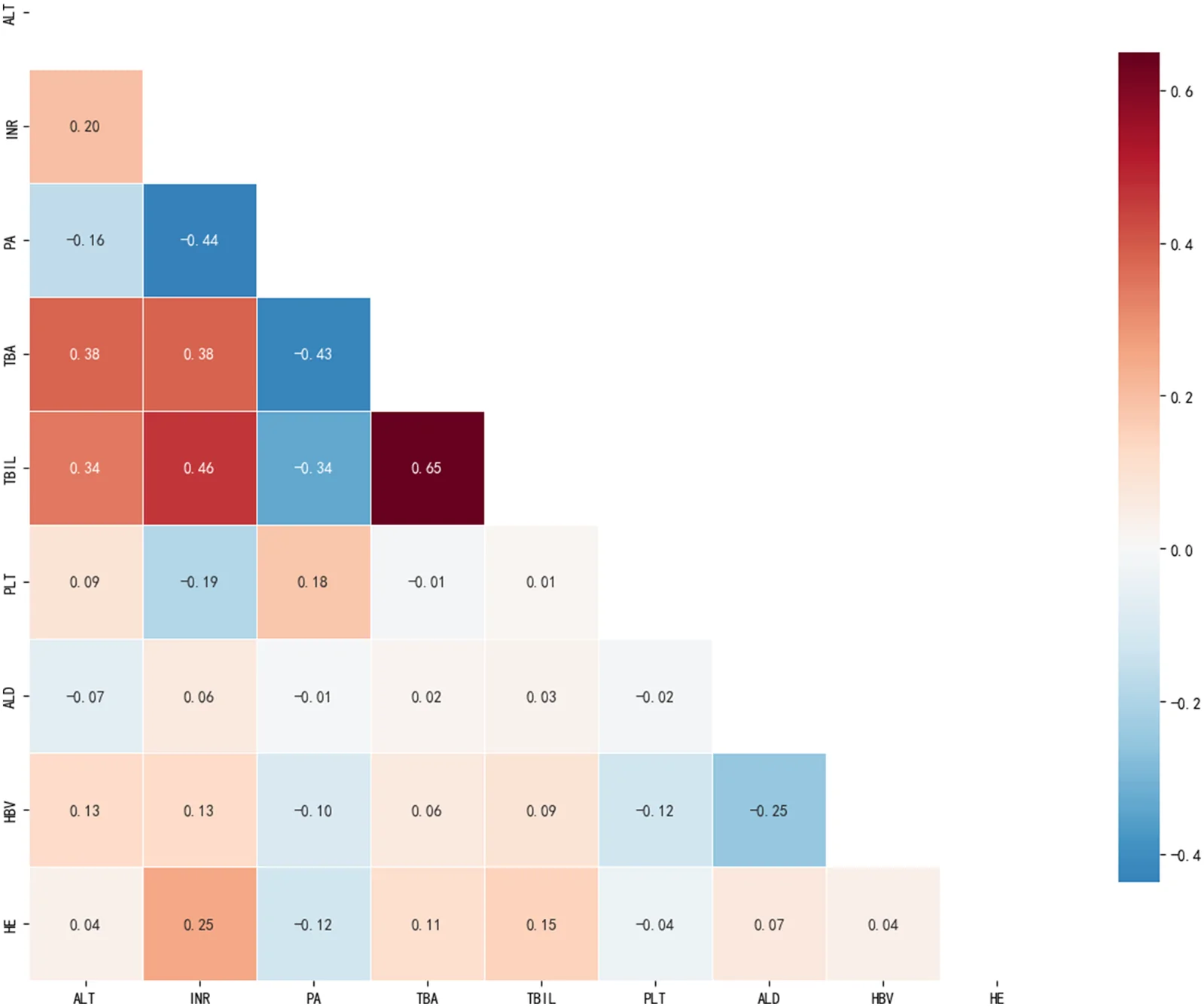
Feature correlation heatmap of Pearson correlation. The diagonal self-correlations (r = 1) carry no information, only the lower off-diagonal triangle is displayed. A threshold of |r| > 0.7 was used to indicate potential multicollinearity.

### Model performance

The selected feature variables were used to train six machine learning models, with hyperparameter tuning performed using Bayesian optimization. A 10-fold cross-validation strategy was applied to evaluate model performance, and the results are presented in Tables 3, 4. In the test set, the AUC–ROC values for XGBoost, RF, LightGBM, GBDT, DT, and LR were 0.945, 0.946, 0.948, 0.947, 0.927, and 0.943, respectively. The corresponding AUC–PR values were 0.667, 0.649, 0.654, 0.640, 0.645, and 0.634 (Figure 4). Table 5 presents the results of the DeLong test, which was used to assess differences in AUC between models. Bonferroni correction was applied to adjust *P*-values for multiple comparisons. The decision tree model demonstrated significantly inferior performance compared with all other models (*P* < 0.05), whereas no statistically significant differences were observed among the remaining models, indicating comparable predictive performance. These results are further detailed in Figure 5**.**

**Table 3:** Predictive performance comparison of the six ML models in the test set.

Model	AUC（95%CI）	Acc	Sen	Spe	PLR	NLR	DOR	F1 Score
DecisionTree	0.927 (0.910,0.942)	0.838	0.901	0.832	5.353	0.119	26.440	0.499
GBDT	0.947 (0.937,0.956)	0.932	0.608	0.964	16.773	0.407	445.940	0.615
LightGBM	0.948 (0.938,0.957)	0.866	0.912	0.861	6.576	0.102	40.837	0.549
LogisticRegression	0.943 (0.933,0.953)	0.839	0.917	0.831	5.431	0.099	26.725	0.505
RandomForest	0.946 (0.935,0.956)	0.870	0.896	0.868	6.781	0.120	44.545	0.554
XGBoost	0.945 (0.935,0.955)	0.871	0.904	0.868	6.856	0.111	45.135	0.558

**Table 4:** Predictive performance comparison of the six ML models in the training set.

Model	AUC	Acc	Sen	Spe	PLR	NLR	DOR	F1 Score
DecisionTree	0.946 (0.939,0.953)	0.853	0.933	0.845	6.007	0.080	32.689	0.532
GBDT	0.956 (0.950,0.962)	0.946	0.685	0.971	23.847	0.325	806.879	0.693
LightGBM	0.960 (0.955,0.965)	0.882	0.954	0.875	7.651	0.052	53.686	0.593
LogisticRegression	0.942 (0.935,0.949)	0.850	0.906	0.845	5.829	0.111	31.668	0.520
RandomForest	0.981 (0.979,0.983)	0.889	0.959	0.882	8.112	0.047	60.515	0.607
XGBoost	0.971 (0.968,0.974)	0.889	0.970	0.881	8.185	0.034	60.865	0.611

**Figure 4:**
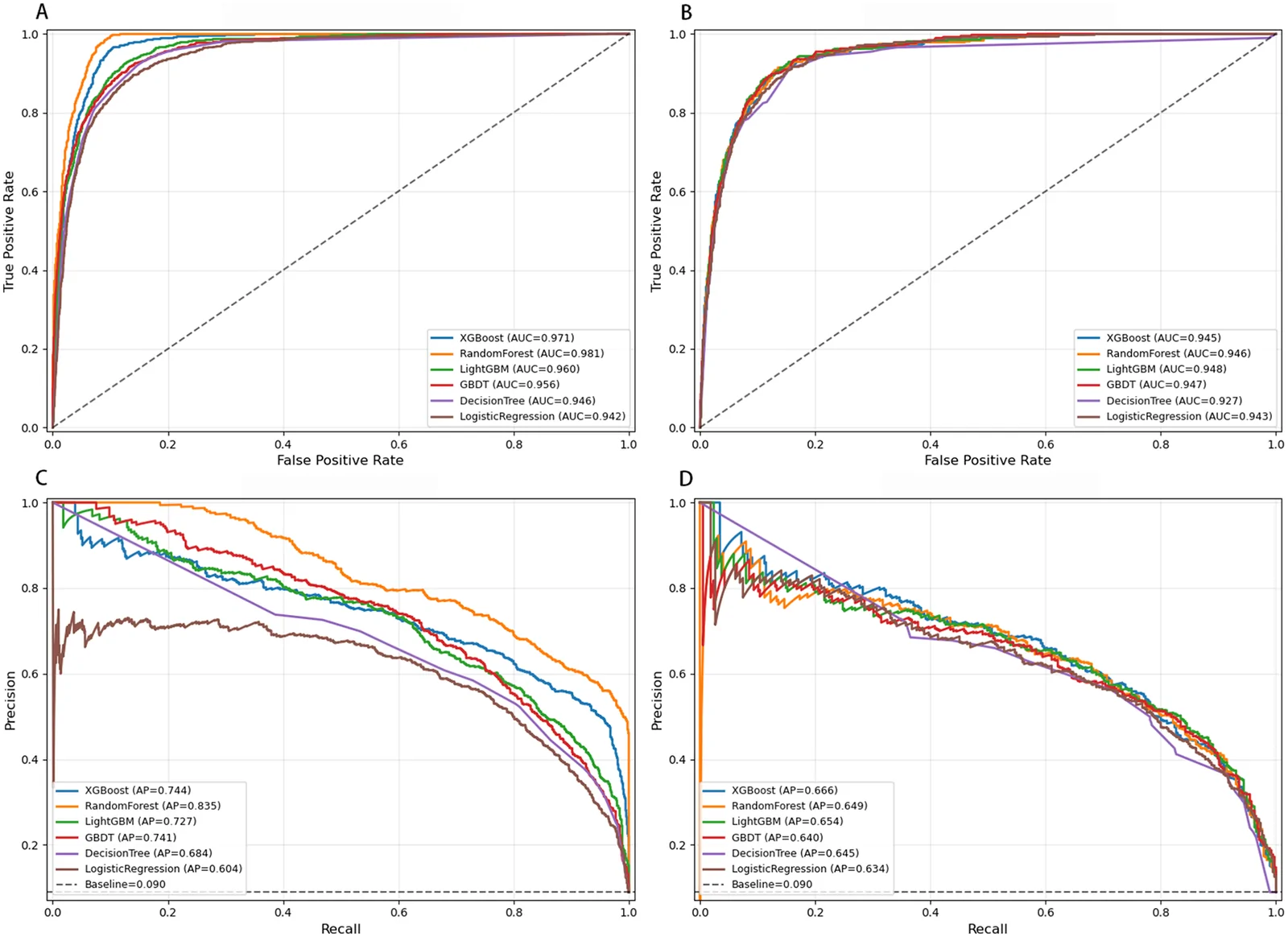
**(A)** ROC curves of the six models in the training set. **(B)** ROC curves of the six models in the test set. **(C)** P-R curves of the six models in the training set. **(D)** P-R curves of the six models in the test set. Extreme gradient boosting (XGBoost), Light gradient boosting machine (LightGBM), Gradient boosting decision tree (GBDT), Receiver operating characteristic (ROC), P-R: Precision-Recall, and Area under the curve (AUC).

**Table 5:** The AUC DeLong test results.

Model 1	Model 2	AUC1	AUC2	*P*-value
XGBoost	DecisionTree	0.945	0.927	0.001
XGBoost	GBDT	0.945	0.947	0.835
XGBoost	LightGBM	0.945	0.948	0.727
XGBoost	LogisticRegression	0.945	0.943	0.700
XGBoost	RandomForest	0.945	0.946	0.918
RandomForest	DecisionTree	0.946	0.927	0.002
RandomForest	GBDT	0.946	0.947	0.846
RandomForest	LightGBM	0.946	0.948	0.718
RandomForest	LogisticRegression	0.946	0.943	0.673
LightGBM	DecisionTree	0.948	0.927	0.019
LightGBM	GBDT	0.948	0.947	0.761
LightGBM	LogisticRegression	0.948	0.943	0.524
GBDT	DecisionTree	0.947	0.927	0.029
GBDT	LogisticRegression	0.947	0.943	0.626
DecisionTree	LogisticRegression	0.927	0.943	0.022

**Figure 5:**
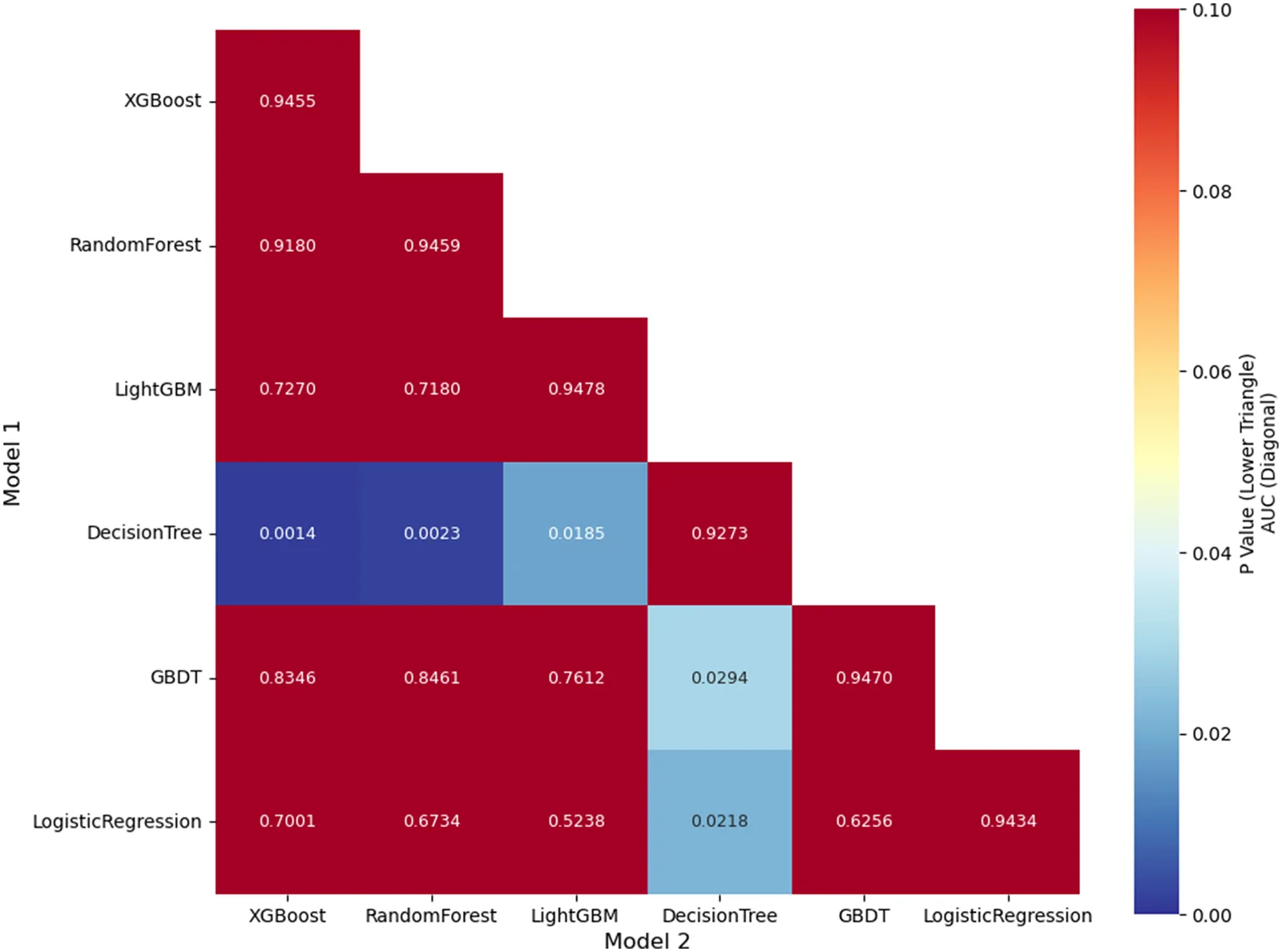
Pairwise comparison of AUC values among six predictive models using deLong test.

Calibration curves for the LightGBM and GBDT models showed good agreement with the diagonal line, indicating strong consistency between predicted and observed probabilities for LC-ACLF patients in both the training and testing sets (Figure 6). DCA demonstrated a substantial net clinical benefit across a range of threshold probabilities for the diagnosis of LC-ACLF (Figure 7). Considering the overall predictive performance of the models, the LightGBM model was selected as the optimal model, with an AUC of 0.948, accuracy of 0.866, sensitivity of 0.912, specificity of 0.861, PLR of 6.576, NLR of 0.102, DOR of 40.837, and F1 score of 0.549.

**Figure 6:**
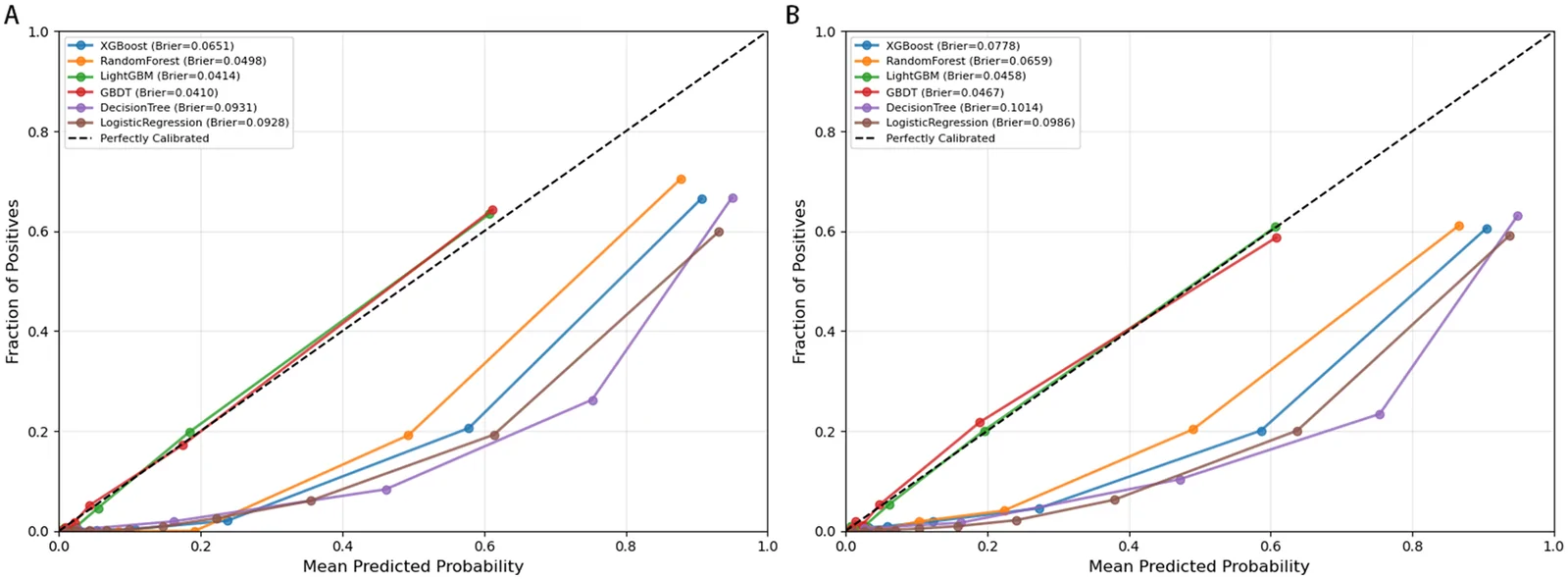
**(A)** Calibration curves of the six models in the training set. **(B)** Calibration curves of the six models in the test set. The diagonal line represents perfect calibration, and each colored line represents a model's calibration curve. The closer the line is to the diagonal, the better the calibration performance of the model.

**Figure 7:**
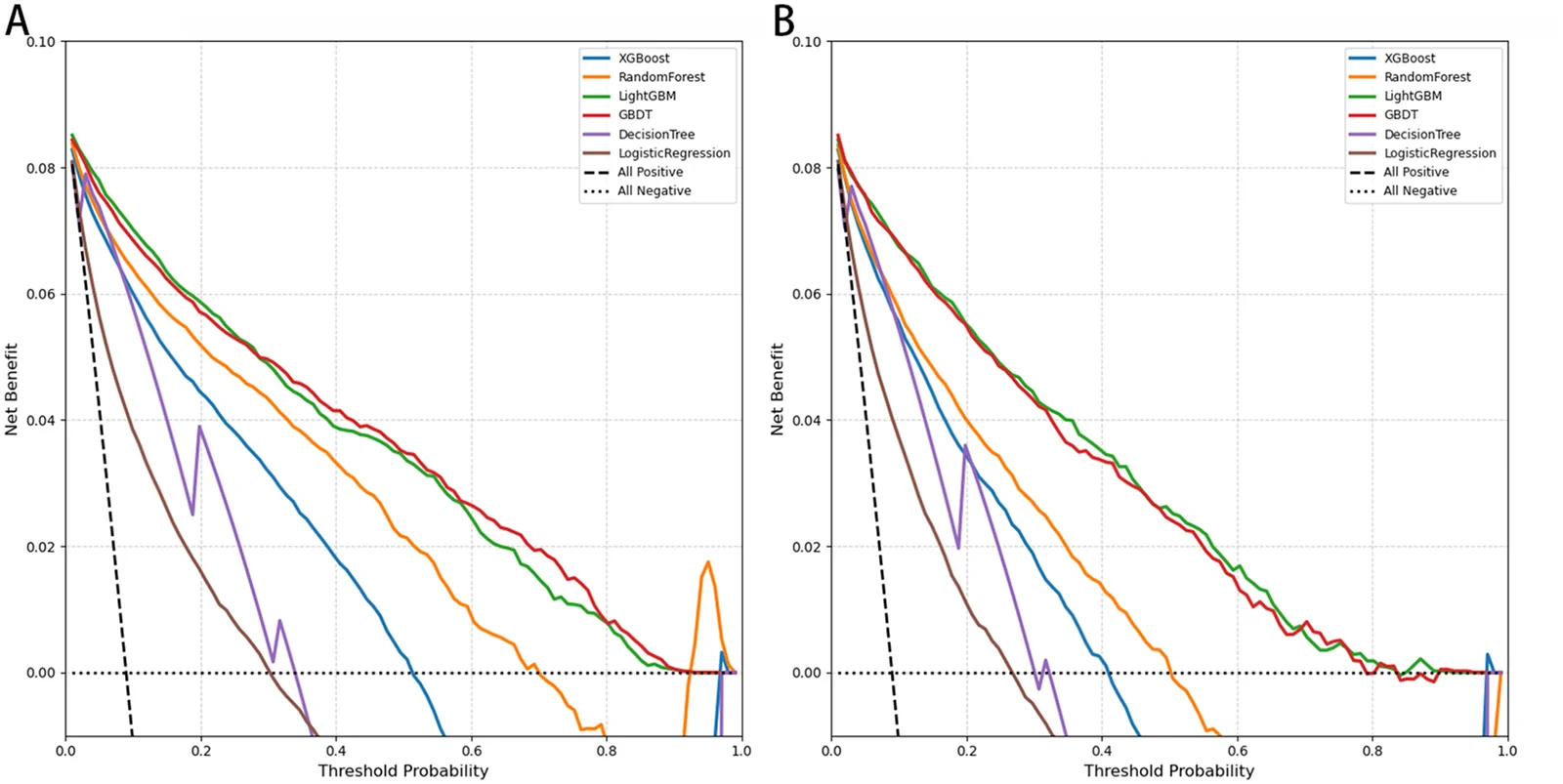
**(A)** Decision curve analyses of the six models in the training set. **(B)** Decision curve analyses of the six models in the test set. The *x*-axis shows the threshold probability, and the *y*-axis denotes the net benefit. The solid black line and the dashed black line represent the net benefit of treating all patients and treating none, respectively. Each colored line shows the net benefit of a model across threshold probabilities.

### SHAP-Based Interpretability of the LightGBM Model

To enhance the interpretability of the LightGBM model, TreeSHAP was applied to quantify the contribution of each feature in the optimal model. High INR, TBIL, ALT, TBA, HE, and ALD shifted SHAP values rightward, indicating increased ACLF risk, whereas high PA and PLT shifted values leftward, indicating protection. The clinical features associated with LC-ACLF, ranked by importance, were INR, TBIL, PA, HBV, ALT, PLT, TBA, HE, and ALD. SHAP-based interpretation revealed that INR, TBIL, and PA were the most influential predictors in the LightGBM model, exhibiting the greatest impact on model output (Figure 8). Individualized explanation analyses demonstrated distinct feature contribution patterns between ACLF and non-ACLF patients. In a representative ACLF case, PA, HBV, and PLT were the primary factors driving increased risk prediction, whereas TBA contributed only modestly. The model generated a prediction score of −4.134, corresponding to a high probability of ACLF. In contrast, for a representative non-ACLF case, lower INR levels were the principal factor associated with reduced risk, resulting in a prediction score of −5.922 and a low predicted probability of ACLF (Figure 9). Analysis of decision trajectories across 20 randomly selected samples demonstrated consistent sequential feature contributions across individuals, indicating stable prediction behavior and robust decision-making patterns (Figure 10). Collectively, these findings enhance the interpretability of the LightGBM model and support its potential clinical utility for identifying patients at high risk of LC-ACLF.

**Figure 8:**
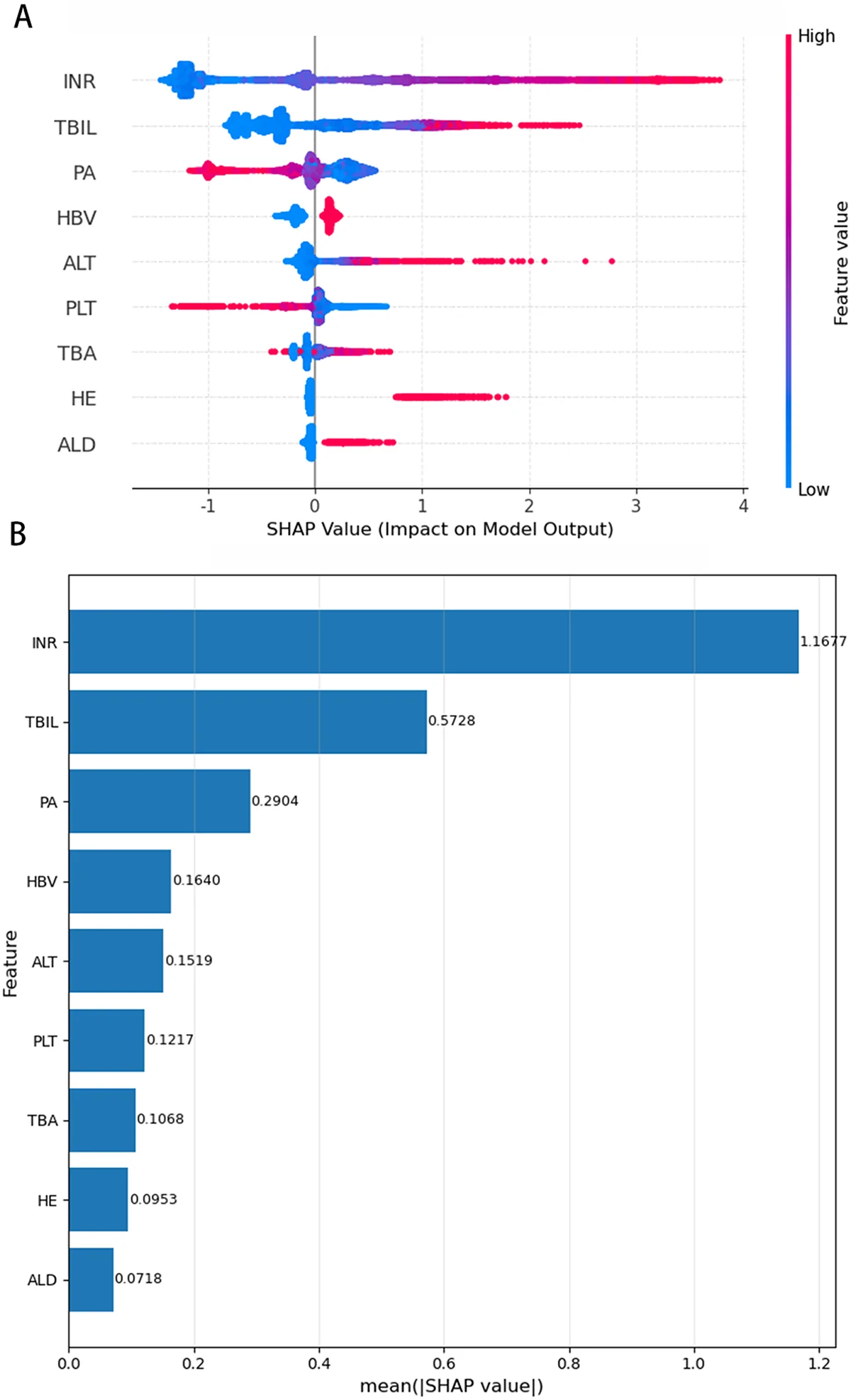
**(A)** SHAP summary plot of the LightGBM model in the test set. Each dot represents one patient. The *x*-axis shows the SHAP value: positive values (right of zero) push the prediction toward ACLF, whereas negative values (left of zero) push it away from ACLF. Dot color indicates the feature value (red, high; blue, low), and features are ranked by mean absolute SHAP value on the *y*-axis. **(B)** Bar plot of global feature importance. Bars represent the mean absolute SHAP value of each feature, quantifying the average magnitude of each feature's contribution to the model.

**Figure 9:**
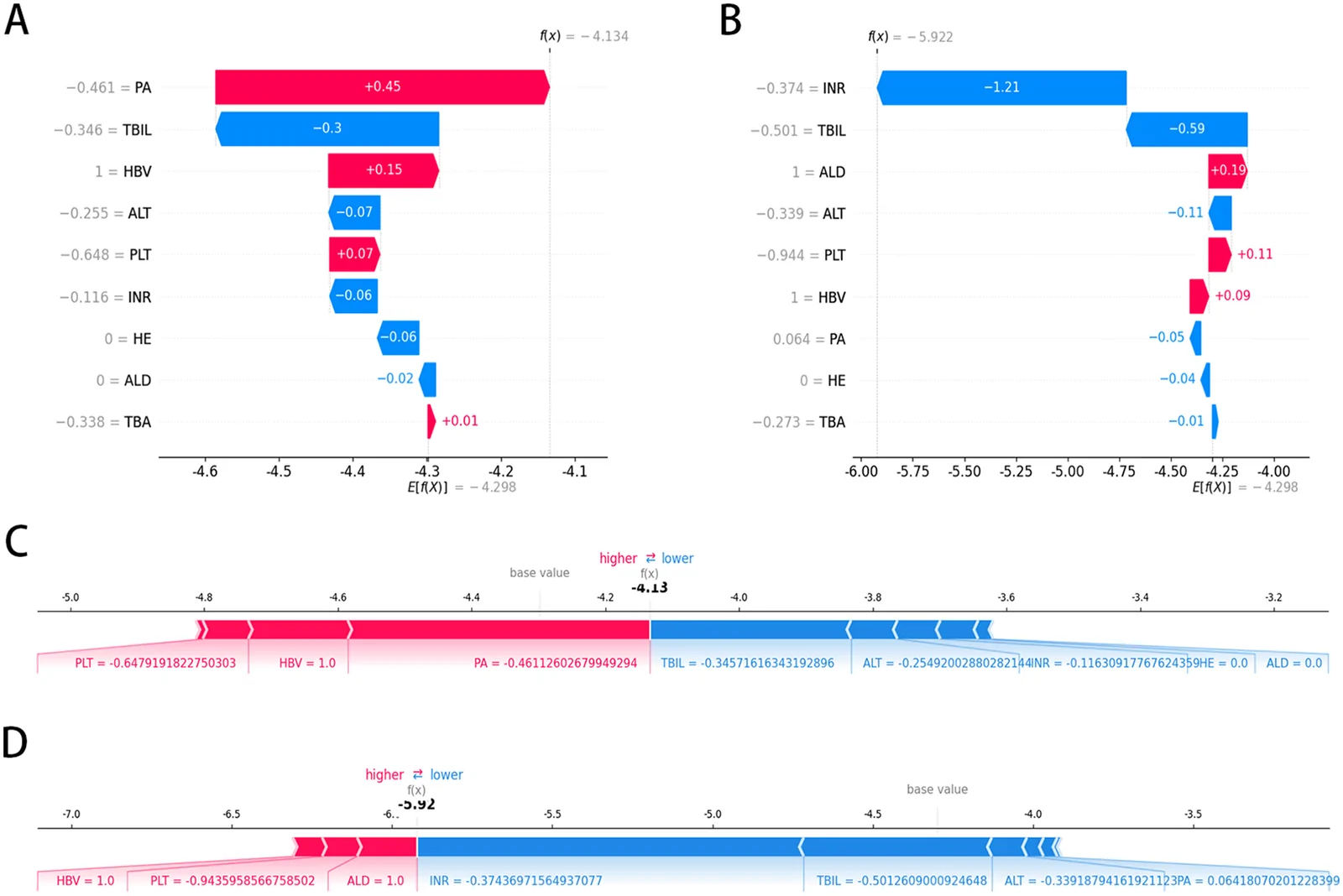
**(A)** SHAP force plot for a positive instance. **(B)** SHAP force plot for a negative instance. **(C)** SHAP waterfall plot for a positive instance. **(D)** SHAP waterfall plot for a negative instance. The force plot depicts how each feature (bar length) contributes to the prediction: red (high value) increases it, while blue (low value) decreases it. The SHAP waterfall plot illustrates the stepwise process by which each feature's SHAP value shifts the model's prediction from the base value to the final output for an individual instance.

**Figure 10:**
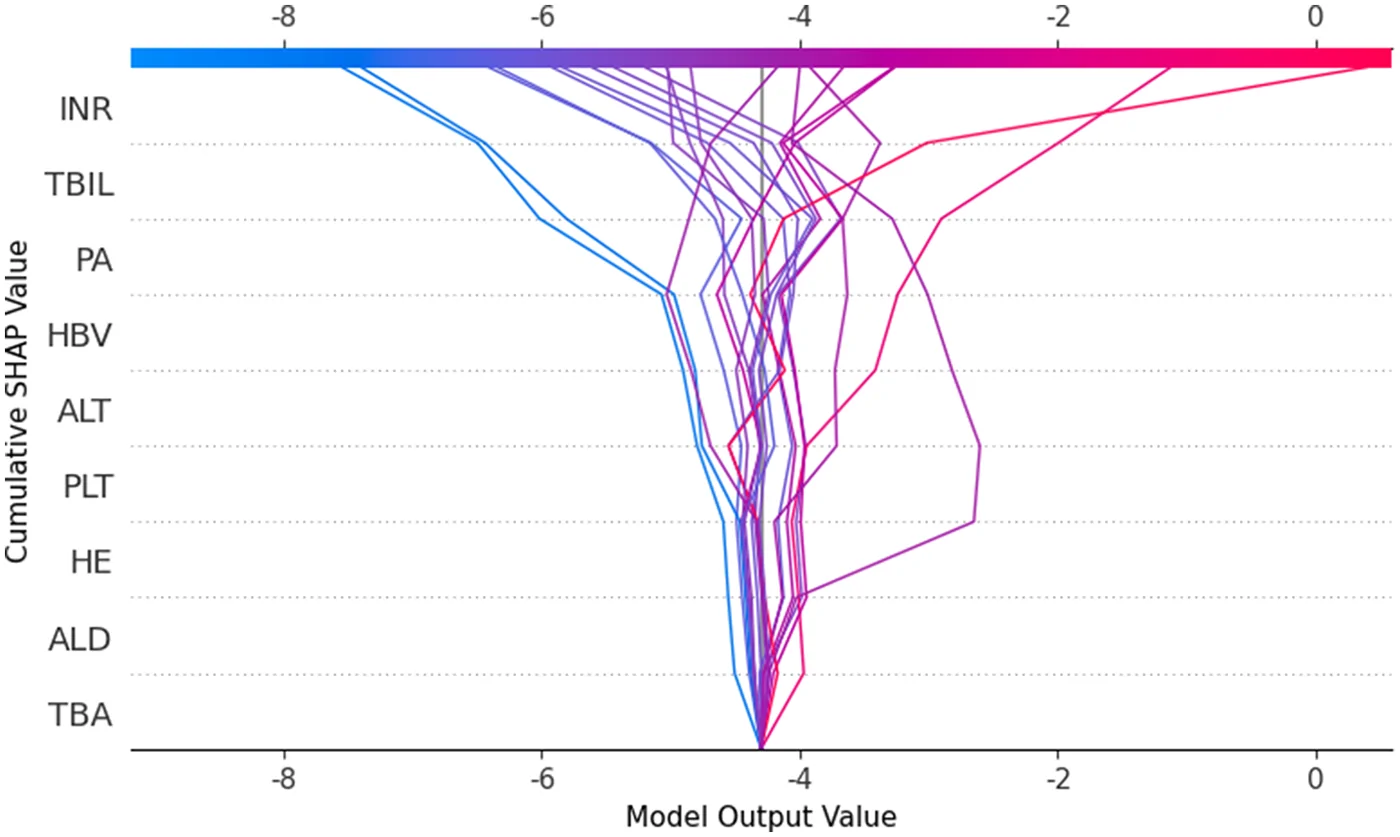
Decision plot for random 20 samples. Each line represents the prediction path of a single sample from the baseline (left) to the final model output (right). An upward shift at a feature indicates that its value increases the model output for that sample, while a downward shift decreases the output.

## Discussion

ACLF is a progressive disease. Due to the absence of obvious clinical manifestations in the early stage, a substantial number of high-risk patients do not receive timely diagnosis and effective treatment, ultimately leading to delayed intervention and poor prognosis (22). Based on a large multicenter retrospective cohort, this study employed multiple machine learning models to develop a tool for the timely identification and risk stratification of ACLF in patients with cirrhosis. The aim was to develop a more accurate diagnostic tool for clinical practice to enable timely detection of ACLF at presentation and provide novel insights into clinical risk stratification and individualized treatment.

In multidimensional risk stratification of ACLF, feature selection is a critical step that largely determines overall model performance. Identifying the most informative variables associated with disease risk from a large number of candidate features remains a major challenge in clinical practice. To address this issue, a logistic-LASSO regression model was applied for feature screening in the present study. This approach effectively reduces overfitting by automatically eliminating irrelevant and redundant variables. Ultimately, it yields a concise, stable, and collinearity-resistant feature set with good interpretability for ACLF risk prediction.

Current studies on ACLF mainly focus on prognostic model development, whereas widely validated tools for disease diagnosis and warning of ACLF remain limited (23, 24). Machine learning algorithms have been widely applied in disease diagnosis and have demonstrated strong predictive performance across various clinical scenarios. In this study, XGBoost, Random Forest, LightGBM, GBDT, and logistic regression all achieved favorable predictive performance, with LightGBM showing the most pronounced advantages. As a lightweight gradient boosting framework, LightGBM offers strong computational efficiency for large-scale, high-dimensional datasets. Its histogram-based decision tree algorithm and leaf-wise growth strategy substantially reduce computational cost, accelerating the training process by several- to tens-fold compared with conventional gradient boosting methods, while also significantly reducing memory consumption. Furthermore, LightGBM incorporates built-in mechanisms for handling class imbalance by automatically computing class weights and integrating them into the loss function. This approach avoids cumbersome data preprocessing steps and further improves the overall efficiency of model development (25).

A total of nine features were selected to build six machine learning models to identify and stratify ACLF risk. The results showed that LightGBM achieved excellent predictive performance on the training set (AUC-ROC = 0.960), and the calibration curve and DCA further confirmed its predictive performance. The validation cohort also demonstrated the model's stability and accuracy (AUC-ROC = 0.948). Compared with previous studies, such as the predictive model proposed by Yuqiang Shen et al. (26), which systematically addressed chronic liver diseases (27), our study provides a more focused and in-depth investigation specifically targeting ACLF prediction in patients with liver cirrhosis. Furthermore, although Jinjin Luo et al. developed a prognostic model for patients with HBV-related liver disease, its predictive performance (AUC = 0.820) was inferior to that of our model. Importantly, the multicenter design and inclusion of patients with multiple etiologies beyond HBV enhance the generalizability of our findings.

Machine learning models are generally regarded as black-box methods, which limits their direct clinical application. To address this limitation, SHAP was introduced for model interpretability. SHAP values were further used to quantify and rank the importance of predictive variables. The SHAP summary plot revealed that nine features, INR, TBIL, PA, HBV, ALT, PLT, TBA, HE, and ALD, had the greatest impact on predictions generated by the LightGBM model. The predictive factors identified in our study were highly consistent with those reported in the early warning model developed by the COSSH cohort for ACLF (28). Notably, prealbumin (PA) ranked highly in the SHAP-based feature importance of our model but has been rarely reported as a key predictor in previous ACLF models. This discrepancy may be attributed to the relatively stable serum concentration of albumin (29), which limits its sensitivity in reflecting the rapid deterioration of hepatic function over the short term. Conversely, PA has a considerably shorter half-life of approximately 1.9 days (30), enabling it to serve as a more sensitive biomarker for acute liver injury and short-term prognostic changes.

Although infection is a well-recognized trigger and driver of ACLF progression, the present model was intentionally designed as a rapid and easily deployable tool based on routinely available admission parameters (31, 32). Infection-specific indicators such as procalcitonin, C-reactive protein, and microbiological cultures (33) were not incorporated owing to their potential reporting delays at admission. While white blood cell count was evaluated as a surrogate marker during feature selection, it was not retained in the final model. Future studies could incorporate more specific infection-related indicators and dynamically monitored laboratory parameters, thereby enhancing the performance of the model. Collectively, these findings provide supportive evidence and valuable insights for the further to establish a truly reliable early-prediction model for ACLF.

To address class imbalance and minimize the impact of data partitioning on model performance, a series of preprocessing steps was performed, including missing-value imputation, standardization of continuous variables, and removal of multicollinear features, before model development. To preserve the original data distribution, the dataset was split into training (70%) and validation (30%) sets using stratified sampling. In addition, class weighting was applied to mitigate the imbalance between positive and negative samples. During the model evaluation phase, the DeLong test was used to assess statistical differences in AUC values across models. Sensitivity and specificity were also jointly considered to evaluate the stability and robustness of the optimal model, thereby ensuring the reliability of the results. Moreover, SHAP was employed to address the “black-box” nature of machine learning models and to visualize the contribution of individual variables. Taken together, this approach provides strong support for the risk stratification of ACLF in patients with liver cirrhosis.

Nonetheless, several limitations should be acknowledged. First, the retrospective design inevitably introduces potential biases. Selection bias may arise from the exclusion of patients with incomplete clinical records, while information bias may occur due to reliance on retrospectively extracted data. Second, although patients with multiple etiologies were included, HBV infection was the predominant etiology in our cohort, reflecting the epidemiological characteristics of cirrhosis in Asia. Third, ALD was defined based on clinical diagnosis without detailed data on alcohol consumption quantity, beverage type, or recent alcohol intake as a potential trigger of ACLF; thus, alcohol-related precipitating events could not be analyzed. Fourth, the inclusion of ascites and hepatic encephalopathy as predictive variables may have inflated model performance, as these indicators are also components of the 2019 APASL ACLF diagnostic criteria. Therefore, further sensitivity analyses that exclude these criterion-based variables are warranted to validate the model's robustness. In addition, although the model was developed and internally validated, external prospective validation is lacking, limiting its generalizability beyond HBV-predominant populations. Additionally, because patient parameters were collected at diagnosis rather than pre-onset, the model facilitates risk stratification at admission but cannot serve as an early-warning tool. Therefore, large-scale, multicenter, prospective external validation studies are warranted to establish a truly reliable early-prediction model for ACLF.

## Conclusion

This study developed and validated a LightGBM-based model for the diagnosis of ACLF using multicenter clinical data. The model demonstrated favorable discriminative performance, with an AUC-ROC of 0.948. SHAP analysis further enhanced model interpretability and identified key predictive factors, including INR, TBIL, and PA. This study was limited by the lack of external prospective validation and by reliance on static clinical variables, which may restrict its generalizability and early-warning performance. Nevertheless, future studies should incorporate dynamic biomarkers and large-scale prospective cohorts to further refine the model. In conclusion, this interpretable machine learning model offers a practical tool for early risk stratification and individualized interventions in patients with ACLF, and may inform the development of a true early-warning model for ACLF.

## Data Availability

The raw data supporting the conclusions of this article will be made available by the authors, without undue reservation.
